# Boric Acid Protects the Uterus and Fallopian Tubes from Cyclophosphamide-Induced Toxicity in a Rat Model

**DOI:** 10.3390/ph17121716

**Published:** 2024-12-19

**Authors:** Enes Karaman, Adem Yavuz

**Affiliations:** 1 Department of Obstetrics and Gynecology, Faculty of Medicine, Nigde Omer Halisdemir University, Nigde 51240, Turkey; 2 School of Health Sciences, Cappadocia University, Nevsehir 50400, Turkey; adem.yavuz@kapadokya.edu.tr

**Keywords:** boric acid, cyclophosphamide, uterus, fallopian tubes, oxidative stress, rat

## Abstract

**Background/Objectives**: Cyclophosphamide (CP) is widely used for treating various cancers and autoimmune diseases, but it causes damage to reproductive organs due to oxidative stress (OS) and inflammation. Boric acid (BA) has antioxidant properties that may help reduce OS, which is critical for preserving uterine functionality, particularly for cancer patients considering pregnancy after cryopreservation. This study aimed to determine whether BA could diminish CP-induced toxicity in the uterus and fallopian tubes (FT) using CP-induced toxicity in a rat model. **Methods**: Forty female Wistar rats, aged 18–20 weeks, were divided into four groups as follows: control, oral BA (OBR), CP, and CP plus OBR (CP + OBR). The toxicity was induced in the CP and CP + OBR groups with an initial dose of 200 mg/kg CP, followed by 8 mg/kg daily for 14 days. Rats in the OBR and CP + OBR groups received 20 mg/kg/day of BA. After the 16-day experiment, tissues were collected for analysis. **Results**: Histopathological and immunohistochemical assessments of IL-6 and HIF-1α expressions were used to evaluate inflammation and OS. The control, OBR, and CP + OBR groups maintained normal tissue features, while the CP group showed epithelial cell shedding, vacuolization, degenerative endometrial glands, lymphocyte infiltration, and reduced collagen fiber density. Elevated HIF-1α and IL-6 expressions in the uterus and FT indicated significant OS and inflammation. **Conclusions**: The study concluded that BA supplementation in CP-treated rats effectively reduced CP-induced uterine and FT damage, suggesting the potential protective role of BA in managing CP-associated toxicity.

## 1. Introduction

Chemotherapy is primarily used to treat malignant tumors, but it can also negatively affect other organs, such as reproductive organs [1,2]. Cyclophosphamide (CP) is one of the alkylating agents that is frequently used in chemotherapy but has high toxicity. CP is one of the oxazaphosphorines classified as nitrogen mustards [3], which are bifunctional AAs [4] and originate from sulfur mustard [5]. CP is a prodrug that is mainly bioactivated (metabolized) in the liver by the cytochrome P450 (CYP) enzyme system, particularly CYP2B6, into several metabolites [6]. The predominant and active metabolite among these is 4-hydroxy-cyclophosphamide (4-OH-CP), which corresponds to more than 80% of the total dose of CP [7]. As 4-OH-CP is subsequently metabolized to aldophosphamide, that is degraded to phosphoramide mustard (PM) and acrolein [6]. PM alkylates the guanine base of DNA at the N7 position and then triggers apoptosis due to the formation of guanine–adenine intrastrand crosslinks [8]. PM can also increase the expression of proapoptotic mRNA and inhibit the expression of antiapoptotic mRNA to promote cell apoptosis [9]. PM mainly exhibits an antineoplastic activity, while the other metabolite acrolein produces highly reactive oxygen species (ROS), interferes with the tissue antioxidant defense system, and is mutagenic to mammalian cells [10,11,12]. Moreover, CP forms phosphotriesters with a relatively high frequency [13], and it is a potent immunosuppressive agent that affects both T- and B-lymphocytes, as it is capable of attenuating both humoral and cell-mediated immune responses [14]. Therefore, CP, which has antimitotic, immunosuppressive, and immunomodulatory effects, is widely used not only in cancer treatment but also in the treatment of autoimmune and immune-mediated diseases as an immunosuppressive agent [15]. However, in the female reproductive system, ovarian follicles are particularly sensitive to the toxic effects of CP [16], and protocols containing CP are four times more likely to result in ovarian failure [17,18]. CP-induced ovarian damage, premature ovarian failure, and infertility are attributed to OS, inflammation, and apoptosis [16]. In addition to its toxic effect on the ovaries, CP can also damage the uterus. CP has been shown to cause a decrease in uterine weight [19], a severe decrease in the number of glandular branches and the amount of stroma [20], an increase in tissue OS, and histological damage [21] in rat models.

Boron is a non-metallic component existing in nature as borax [sodium tetraborate] and BA. The prevailing shape of boron in plasma is BA [22]. The pharmacokinetics of BA in people and rodents are astoundingly comparable; following oral administration, BA is exceedingly retained, is not metabolized, and is mainly excreted in urine [23]. The biochemical mechanism of BA is not yet fully understood. However, two hypotheses have been proposed regarding the biochemical and physiological functions of BA in animals, including humans. The first hypothesis suggests that BA may play a role in cell membrane functions influencing the response to hormone action, transmembrane signaling, and the transmembrane movement of regulatory ions [24], while the second hypothesis suggests that BA may act as a metabolic regulator in various enzymatic systems [25]. It has been reported that boric acid given in non-lethal doses with the standard diet can reduce lipid peroxidation, and therefore has an antioxidant effect [26]. In the experimental ovarian ischemia and reperfusion model, it has a therapeutic effect by decreasing the oxidative stress parameters and autophagy biomarkers [27]. In different studies where oxidative stress was induced with CP, BA has shown the effect of reducing oxidative stress and apoptotic markers in the bladder and testis [28,29].

BA improves antioxidant defense mechanisms [26], hypoxia inducible factor-1α (HIF-1α) mediates adaptive responses to OS [30], and interleukin-6 (IL-6) is a key immunomodulatory cytokine that affects the pathogenesis of chronic inflammatory conditions [31]. Based on these findings, it may be hypothesized that BA supplementation amid CP treatment may have a defensive impact against OS-induced harm within the uterus and FT by balancing HIF-1α and IL-6. In our study, we aimed to examine the conceivable impact of BA supplementation on the expressions of HIF-1α and IL-6 within the uterus and FT in CP-induced toxicity in a rat model.

## 2. Results

### 2.1. Histopathology

**Figure 1:**
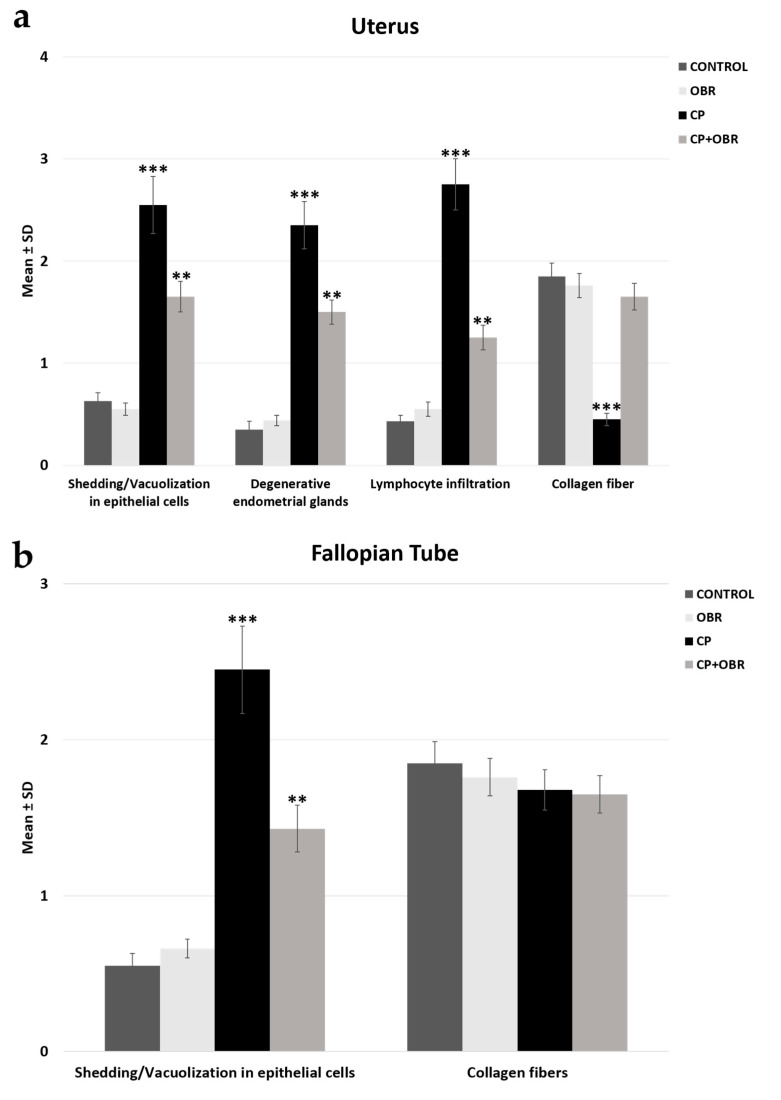
The evaluation of histopathological parameters, shedding/vacuolization in epithelial cells, degenerative endometrial glands, lymphocyte infiltration, and collagen fiber of the uterus (**a**) and fallopian tube (**b**) across the control, OBR, CP, and CP + OBR groups. ** *p* < 0.01, *** *p* < 0.001.

**Figure 2:**
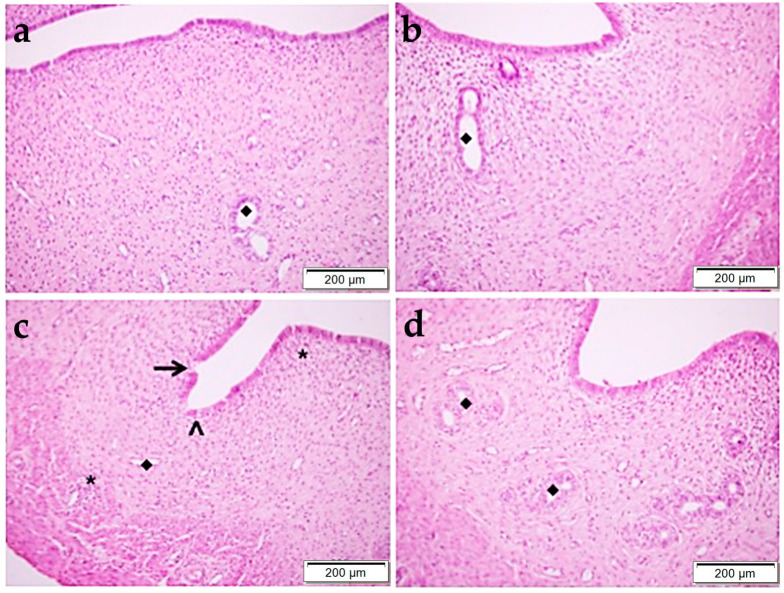
Histopathological examination of the uterus by H&E staining in control (**a**), OBR (**b**), CP (**c**), and CP + OBR (**d**) groups. Scale bars: 200 µm. Endometrial glands (◆), shedding epithelial cells (arrow), vacuolization in epithelial cells (arrowhead), lymphocyte infiltration areas (*).

H&E staining were used to determine the histopathological changes (Figure 1 and Figure 2). In the control group, the endometrial layer exhibited its normal histological structure through the presence of single-layered columnar epithelial cells, blood vessels, and endometrial glands with smooth luminal structures in the connective tissue (Figure 2a). In the OBR group, the histological structure of the uterus was similar to the control group (Figure 2b). But, in the CP-administered group, shedding and vacuolization in epithelial cells, degenerative endometrial glands, and lymphocyte infiltration areas in the connective tissue were observed. The tissue damage was reduced by administering BA together with the CP, and the uterus structure was found to be similar to the control group (Figure 2d).

**Figure 3:**
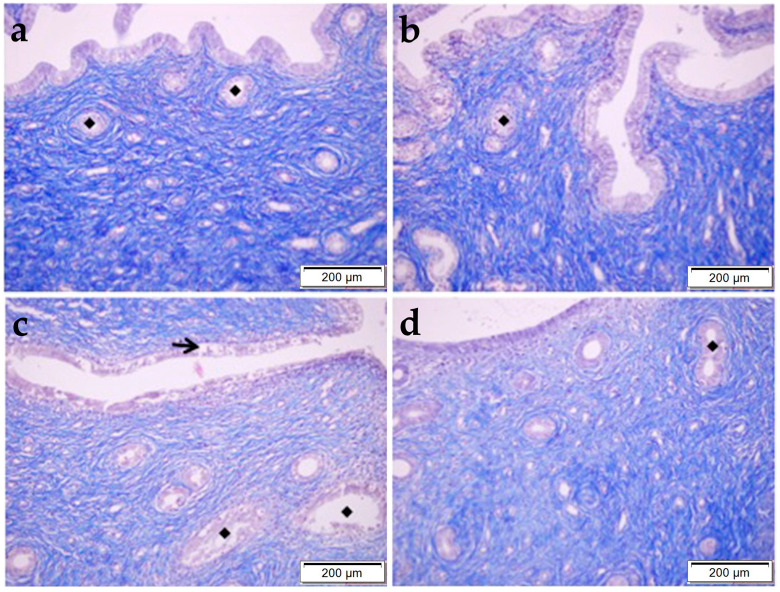
Histopathological examination of the uterus by MT staining in control (**a**), OBR (**b**), CP (**c**), and CP + OBR (**d**) groups. Scale bars: 200 µm. Endometrial glands (◆), shedding epithelial cells (arrow).

The MT procedure was applied to the sections to evaluate the collagen fiber density. Decreased collagen fiber density was particularly observed in the CP group compared to the control group. In the CP + OBR group, the staining intensity was higher than in the CP group (Figure 3).

**Figure 4:**
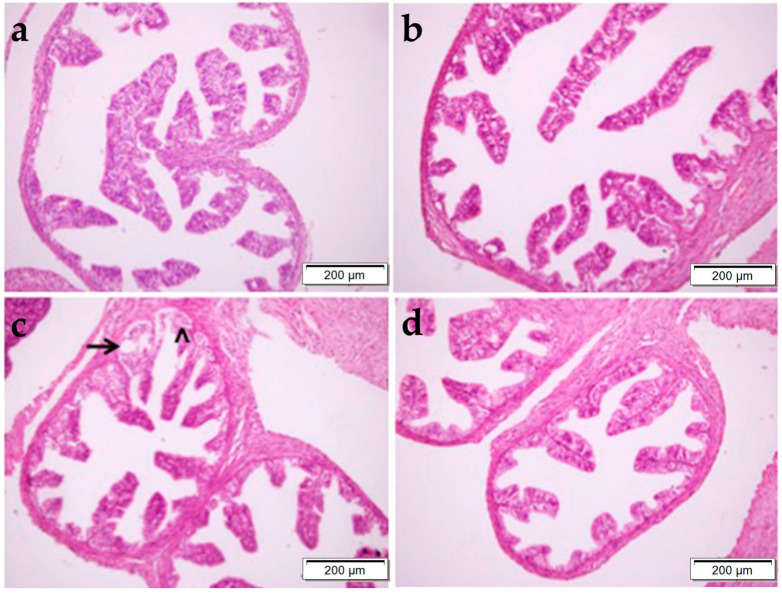
Histopathological examination of the fallopian tube by H&E staining in control (**a**), OBR (**b**), CP (**c**), and CP + OBR (**d**) groups. Scale bars: 200 µm. Shedding epithelial cells (arrow), vacuolization in epithelial cells (arrowhead).

**Figure 5:**
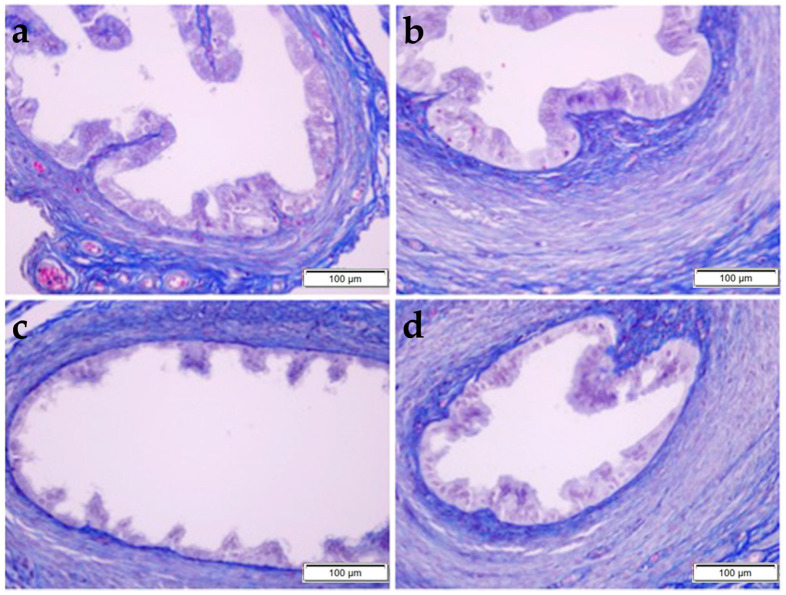
Histopathological examination of the fallopian tube by MT staining in control (**a**), OBR (**b**), CP (**c**), and CP + OBR (**d**) groups. Scale bars: 100 µm.

In Figure 4, it was determined that the normal histological structure of the FT was preserved in the control, OBR, and CP + OBR groups, whereas epithelial cell shedding and vacuolization were noted in the CP group. Also, in the MT-stained sections, no clinical difference was observed in terms of collagen fiber density and fibrosis (Figure 5).

### 2.2. Immunohistochemical Analysis

**Figure 6:**
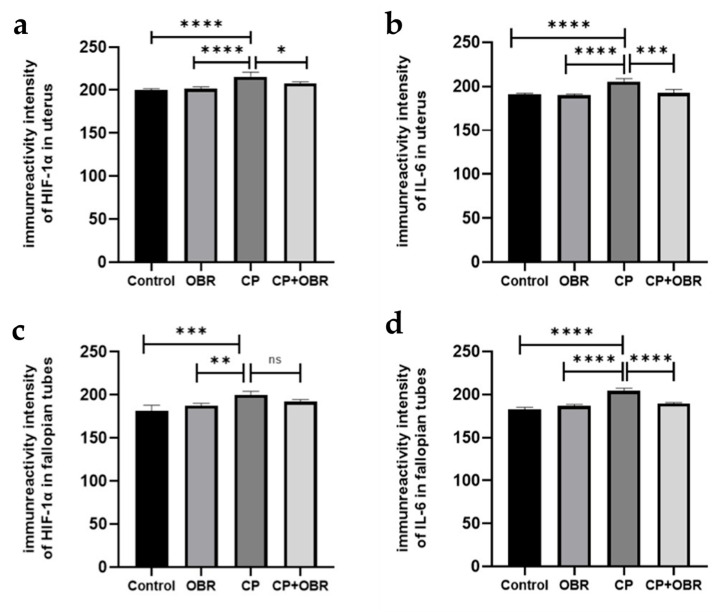
The statistical analysis of HIF-1α (**a**,**c**) and IL-6 (**b**,**d**) immunoreactivity in the uterus and fallopian tubes from the control, OBR, CP, and CP + OBR groups. * *p* < 0.05, ** *p* < 0.01, *** *p* < 0.001, **** *p* < 0.0001; ns, not significant/*p* < 0.05.

Expressions of HIF1α and IL-6 molecules were evaluated using the immunohistochemistry method (Figure 6). 

**Figure 7:**
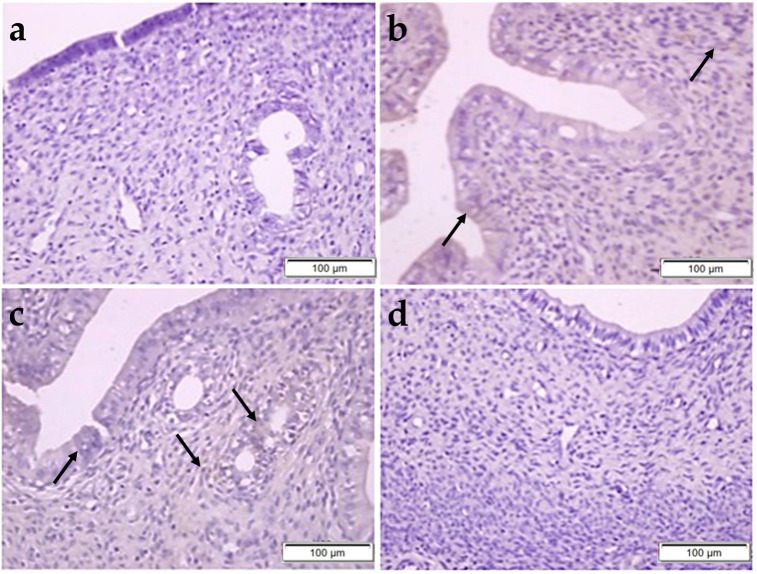
The immunoreactivity of HIF-1α in rat uterus from the control (**a**), OBR (**b**), CP (**c**), and CP + OBR (**d**) groups. Scale bars: 100 µm. Immunopositive cells (arrows).

In uterine sections, we found that the CP group had a statistically higher HIF-1α expression compared to the other groups (CP–control; *p* = 0.0046, CP–OBR; *p* = 0.0116, CP–CP + OBR; *p* < 0.05), which is expected to increase due to the induction of proinflammatory cytokines (Figure 7).

**Figure 8:**
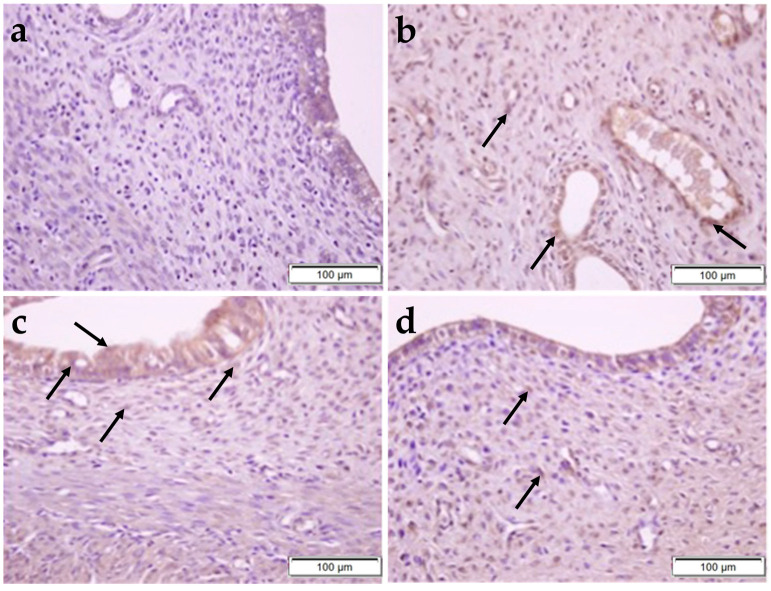
The immunoreactivity of IL-6 in rat uterus from the control (**a**), OBR (**b**), CP (**c**), and CP + OBR (**d**) groups. Scale bars: 100 µm. Immunopositive cells (arrows).

Additionally, in uterine sections, the CP group’s expression of the proinflammatory cytokine IL-6 was statistically significantly higher than that of the other groups. OBR application in the uterus was found to reduce IL-6 expression, which was the highest in the CP group compared to the other groups (Figure 8). The results were statistically significant (CP–control; *p* < 0.0001, CP–OBR; *p* < 0.0001, CP–CP + OBR; *p* = 0.0002).

**Figure 9:**
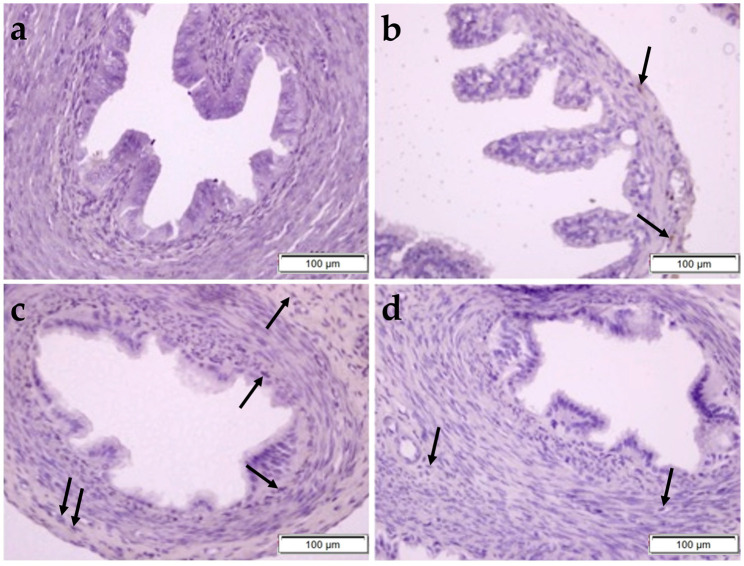
The immunoreactivity of HIF-1α in rat fallopian tubes from the control (**a**), OBR (**b**), CP (**c**), and CP + OBR (**d**) groups. Scale bars: 100 µm. Immunopositive cells (arrows).

HIF-1α expression was found to be significantly higher in the sections of the FT tissue of the CP group compared to the control and OBR groups, but the difference between the CP + OBR group was not significant (CP–control; *p* = 0.0002, CP–OBR; *p* = 0.0066, CP–CP + OBR; *p* = 0.1294) (Figure 9).

**Figure 10:**
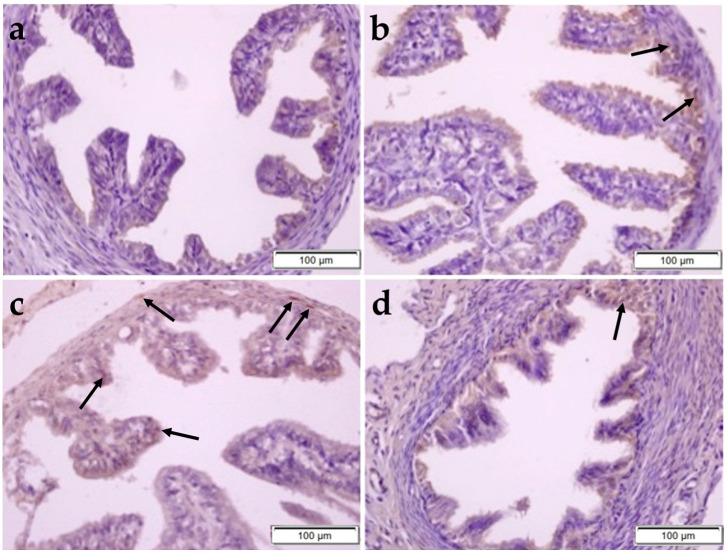
The immunoreactivity of IL-6 in rat fallopian tubes from the control (**a**), OBR (**b**), CP (**c**), and CP + OBR (**d**) groups. Scale bars: 100 µm. Immunopositive cells (arrows).

IL-6 expression was higher in the sections of the CP group compared to the others, and the results were statistically significant. It was shown that the increase in IL-6 level due to CP decreased after OBR application (CP–control; *p* < 0.0001, CP–OBR; *p* < 0.0001, CP–CP + OBR; *p* < 0.0001) (Figure 10).

## 3. Discussion

The objective of this study was to ascertain whether oral BA supplementation, administered in conjunction with CP, exerts a protective effect against the toxic effects of CP on the uterus and FT in a CP-induced toxicity model in rats. The findings of this study indicate that oral BA supplementation given with CP protected against CP-induced endometrial gland degeneration and collagen fiber loss. These previously unreported protective effects of BA against the toxic effects of CP on the uterus may be significant in terms of preserving female fertility in a group of patients who underwent oocyte or embryo cryopreservation before treatment with CP-containing protocols.

Despite a comprehensive understanding of CP-induced ovarian toxicity [16], there is limited data on the effects of CP on the uterus. Yılmaz et al. [21] observed that the administration of CP via intraperitoneal injection at a dosage of 200 mg/kg on day 1, followed by 8 mg/kg/day for 14 days, resulted in a significant increase in vascular congestion, inflammatory cells in the lumen, epithelial degeneration, vacuoles in epithelial cells, and dilated endometrial glands in the endometrium layer of the uterus of rats. Bulbul et al. [32] observed that CP administered as a single intraperitoneal dose of 100 mg/kg caused the degeneration of cubic epithelial cells forming the epithelium of glands, a decrease in collagen fibers in the connective tissue, and a dilatation in the stromal blood vessels in the uterine tissue of rats 16 days after the administration. Furthermore, Pascuali et al. observed that a significant decrease in the number of glandular branches and the amount of stroma were observed in the CP-treated rat uterus in a premature ovarian failure (POF) model [20]. In our study, shedding in the luminal epithelial cells, vacuolization in the epithelial cells, and degenerated endometrial glands were observed in the endometrium layer of the uterus of rats in the CP group. Also, we observed lymphocyte infiltration and a decrease in collagen fibers density in the connective tissue of the uterus. The current study yielded results consistent with those of previous studies [21,32] in demonstrating the toxic effects of CP on the endometrial lining of the uterus. Thus, it has been shown that the drug dose and duration of exposure cause tissue-level damage in the uterus.

The current study also assessed the impact of CP on the FT. The results demonstrated that CP induced epithelial cell shedding and vacuolisation in the FT, a finding that was similar to that observed in the uterine epithelium. However, CP did not affect the density of collagen fibers in the FT. A previous study evaluated whether female reproductive tract organs from the same mouse exhibit a comparable inflammatory state and determined that discrepancies in inflammatory activity scores are predominantly tissue-specific. Furthermore, uterine fibroblasts predominantly receive anti-inflammatory signals, while the FT and ovaries exhibit a combination of anti-inflammatory and proinflammatory signals [33]. It can therefore be postulated that CP may exert differing effects on fibroblasts in the uterus and FT, specifically in regard to inflammation and the process of remodeling.

It has been reported that hypoxia, which represents a pathological process that causes abnormal changes in tissue metabolism, function, and morphological structure due to insufficient oxygen supply, is accompanied by an increased production of ROS and thus provokes OS [34]. Many studies have demonstrated that CP cause OS in a dose- and time-dependent manner [21,35,36,37]. Previous studies have shown that supplementing rats with BA at different daily doses and treatment durations can reduce lipid peroxidation and improve antioxidant defense mechanisms [26,29,35], mitigate ovarian ischemia/reperfusion and DNA damage [33], protect the testis and bladder [28] from CP-induced damage by its antioxidant and antiapoptotic properties [29], and mitigate CP-induced lipid peroxidation and genotoxicity [35]. Moreover, antioxidant therapy has been shown to alleviate uterine OS and to reduce the uterine [21] and endometrial [32] tissue damage associated with the systemic CP treatment in rats. The transcription factor HIF-1α mediates adaptive responses to OS by nuclear translocation and the regulation of gene expression [30]. The results of our study indicated that HIF-1α expression is significantly higher in the uterus in the CP group compared to other groups and in the FT compared to the control and OBR groups. However, while HIF-1α expression was higher in FT sections compared to the CP + OBR group, the difference was not statistically significant. It is notable that the CP-induced tissue damage observed in the uterus and FT in the CP group was not observed in the CP + OBR group. Based on these findings, we can conclude that CP treatment induced hypoxic conditions in uterine and FT tissues, leading to an increase in HIF-1α expression. In addition, the addition of BA to CP treatment caused a significant decrease in HIF-1α expression in uterine tissue, whereas this decrease was not statistically significant in FT tissue. This observed trend of decreased HIF-1α levels suggests that BA may play an important role in preventing CP-induced tissue damage, especially in the uterus.

IL-6 is a crucial immunomodulatory cytokine that influences the development of a range of diseases, including chronic inflammatory conditions [31]. Despite the observed increase in inflammatory processes during the postmenopausal period, serum IL-6 levels remain unaltered in ovariectomized rats [38]. It has been demonstrated that IL-6, which is elevated in damaged ovarian tissue following experimental ischemia/reperfusion, undergoes a biochemical reduction following BA administration [39]. Additionally, NF-kappaB may be activated by hypoxia and move to the nucleus, which would increase IL-6 transcription and expression [40]. The results of our investigation into the toxicity model in rats demonstrated that CP treatment alone led to a notable elevation in IL-6 immune expression in both uterine and FT tissues when compared to the other experimental groups. In contrast, the combination of CP treatment with BA resulted in a pronounced reduction in IL-6 immune expression in both types of tissues. Based on these findings, it can be proposed that BA may also have a protective role against CP-induced damage in the uterus and FT by regulating the immune system.

In summary, systemic CP treatment in rats causes damage to uterine [21] and endometrial [32] tissues. Although it is not possible to determine whether the uterine myometrium will be able to fulfill its functions before pregnancy, degenerated uterine glands have been shown to exhibit functional abnormalities, including an increase in ciliated cells, atypical mucus production, reduced expression of epidermal growth factor, and abnormal patterns of proliferation [41]. The data obtained from studies on different tissues in the literature demonstrate that CP causes OS [21,35,36,37]. On the other hand, BA supplementation has been demonstrated to enhance antioxidant defense systems [26,29,35] and provide protection against tissue damage caused by CP [30,31]. Furthermore, antioxidant treatment has been demonstrated to reduce uterine [21] and endometrial [32] tissue damage associated with systemic CP treatment in rats. In humans, one week of boron supplementation results in a statistically significant decrease in plasma tumor necrosis factor-alpha levels, as well as a remarkable decrease in high-sensitivity C-reactive protein and proinflammatory IL-6 levels [42]. The results of our study in the toxicity model in rats demonstrate that BA, when added to CP treatment, significantly reduced CP-induced damage to the uterus and FT, probably by improving the hypoxic environment that prevents OS exposure and regulating inflammation. These findings should be supported by studies in humans. However, our findings provide evidence that the addition of BA supplementation to CP treatment may represent a promising strategy for fertility preservation in female cancer patients undergoing oocyte and/or embryo cryopreservation prior to CP treatment.

## 4. Materials and Methods

### 4.1. Chemicals and Antibodies

CP and BA were purchased from Merck (Merck, Darmstadt, Germany; CAS number 6055-19-2 and CAS number 10043-35-3). For the immunohistochemistry, the anti-HIF-1α mouse monoclonal antibody (sc-53546, Santa Cruz, CA, USA; mouse monoclonal with the clone number H1alpha 67; 1:100 dilution), and anti-IL-6 polyclonal antibody (bs-0782R, Bioss, Woburn, MA, USA; rabbit polyclonal anti-mouse IL-6 antibody, 1:250 dilution) were used. The Ultra Vision Detection System, a streptavidin-biotin immunoenzymatic detection system used in immunohistochemical staining, was obtained from Thermo Fisher Scientific (Thermo Fisher Scientific, Waltham, MA, USA).

### 4.2. Animals

Experimental procedures were approved by the Nigde Omer Halisdemir University Local Ethics Committee on Animal Experimentation (date: 31 May 2024; approval number: 2024/08), and experiments were conducted according to the Guide for the Care and Use of Laboratory Animals published by NIH (1996). The sample size for each group was calculated as 10 with an 80% power, 0.05 statistical significance level, and 0.80 effect size (cohen’s d) using the G*Power version 3.1.9.2 software (Universität Kiel, Kiel, Germany). The study included forty female Wistar-Albino rats, aged 18-20 weeks and weighing between 180 and 210 g. The rats were kept in standard bedding cages at a room temperature between 21 and 23 °C. Rats were kept on a constant light schedule from 7 a.m. to 7 p.m., with sufficient food and water available ad libitum.

### 4.3. Experimental Protocol

Rats were randomly divided into four groups as follows: control, OBR, CP, and CP + OBR (Table 1). The control group was given no agent whereas BA (20 mg/kg/day) was administered orally to the OBR group for 15 days. The experimental toxicity model was induced by CP administration in the CP and CP + OBR groups; CP was administered intraperitoneally to female rats at a dosage of 200 mg/kg/day on the first day, followed by 8 mg/kg/day for 14 consecutive days [43]. Twenty-four hours after the final BA administration (on day 16), rats received 50 mg/kg of ketamine and 5 mg/kg of xylazine, the uterus and FT were dissected via laparotomy, and the rats were decapitated.

### 4.4. Histological Procedure

Samples obtained from the uterus and FT of rats were fixed in 10% formaldehyde solution. Next, the tissue samples were sliced into small sections, dehydrated using a series of increasing ethanol concentrations (70%, 90%, 96%, and 100%), cleared in xylene, and then embedded in paraffin blocks. The tissue samples were cut in thicknesses of 5 μm and stained with hematoxylin-eosin (H&E) and Masson trichrome (MT). They were then examined using a light microscope (BX51, Olympus, Tokyo, Japan). The histopathological evaluation was performed with the parameters of shedding or vacuolization in epithelial cells, degenerative endometrial glands, lymphocyte infiltration, and collagen fiber in the uterus and FT tissue samples. The parameters were scored as 0, none; 1, mild; 2, moderate; and 3, severe [19]. The results were analyzed statistically.

### 4.5. Immunohistochemical Procedure

In order to determine alterations in HIF-1α and IL-6 immunoreactivity within the uterus and FT, the avidin-biotin-peroxidase method was used in accordance with the instructions provided by the manufacturers (Santa Cruz Biotechnology, Dallas, TX, USA, and Bioss Inc., Woburn, MA, USA). The sections were briefly washed in deionized water, rehydrated, and put in a microwave for five minutes at 95 °C in 0.01 M sodium citrate buffer (pH 6.0) for antigen recovery. Hence, the sections were washed in phosphate-buffered saline (PBS). They were treated with 3% hydrogen peroxide to inhibit endogenous peroxidase activity. The subsequent steps were performed using the Ultra Vision Detection System staining kit (Thermo Fisher Scientific, Waltham, MA, USA), in accordance with the manufacturer’s instructions. In order to avoid non-specific staining, the sections were treated in the Ultra V block for 10 min, then incubated with primary antibodies, anti-HIF-1α and anti-IL-6, overnight at 4 °C. After washing with PBS, they were treated with biotin-streptavidin hydrogen peroxidase secondary antibodies for 10 min, and they were washed in PBS. Sections were stained with 3,3′-diaminobenzidine tetrahydrochloride (DAB) for 10 min to make immunoreactivity visible. And Gill’s hematoxylin procedure was performed for counterstaining. After alcohol and xylene applications, the sections were mounted. In order to acquire immunohistochemical images, a light microscope (BX51, Olympus, Tokyo, Japan) was utilized. The immunoreactivities of HIF-1α and IL-6 were determined using the ImageJ.JS v0.5.8 Software (National Institutes of Health, Bethesda, MD, USA) on ten randomly selected fields.

### 4.6. Statistical Analysis

The data were analyzed using GraphPad Prism software (version 8.0d, GraphPad Software Inc., San Diego, CA, USA). The normal distribution was verified through the implementation of the Shapiro–Wilk test, and the significance of the findings was subsequently analyzed through the utilization of one-way ANOVA. In order to make comparisons between multiple groups, the Bonferroni multiple comparisons test was employed. A *p*-value of less than 0.05 was considered statistically significant, with a sample size of *n* = 10 for all groups.

## 5. Conclusions

In conclusion, the results of our study indicate that BA supplementation in conjunction with CP treatment markedly attenuates CP-induced damage in the uterus and FT. It has also been shown that boric acid prevents the oxidative stress caused by the drug in the acute phase. Future research is needed to clarify the protective effects of BA on the uterus in women undergoing chemotherapy who wish to preserve their fertility.

## Figures and Tables

**Table 1:** Experimental groups.

Groups	Agent	Dosage	Gender	*n*
**Group I: Control**	No	No	Female	10
**Group II: OBR**	Boric acid (*Orally*)	20 mg/kg/day (for 15 days)	Female	10
**Group III: CP**	Cyclophosphamide (*i.p.*)	200 mg/kg/day (only first day) and 8 mg/kg/day (for 14 days)	Female	10
**Group IV: CP + OBR**	Cyclophosphamide (*i.p.*)	200 mg/kg/day (only first day) and 8 mg/kg/day (for 14 days)	Female	10
	Boric acid (*Orally*)	20 mg/kg/day (for 15 days)		

*n: Number of rats.*

## Data Availability

The raw data supporting the conclusions of this article will be made available by the authors upon request.
